# Three-Dimensional Calcium Alginate Hydrogel Assembly via TiOPc-Based Light-Induced Controllable Electrodeposition

**DOI:** 10.3390/mi8060192

**Published:** 2017-06-19

**Authors:** Yang Liu, Cong Wu, Hok Sum Sam Lai, Yan Ting Liu, Wen Jung Li, Ya Jing Shen

**Affiliations:** 1 Department of Mechanical and Biomedical Engineering, City University of Hong Kong, Hong Kong, China; yliu565@cityu.edu.hk (Y.L.); congwuandy@gmail.com (C.W.); samlai5-c@my.cityu.edu.hk (H.S.S.L.); yantinliu2-c@my.cityu.edu.hk (Y.T.L.); wenjli@cityu.edu.hk (W.J.L.); 2 Shenzhen Research Institute, City University of Hong Kong, Shenzhen 518057, China

**Keywords:** three-dimensional (3D) hydrogel assembly, TiOPc, alginate hydrogel, light-induced electrodeposition

## Abstract

Artificial reconstruction of three-dimensional (3D) hydrogel microstructures would greatly contribute to tissue assembly in vitro, and has been widely applied in tissue engineering and drug screening. Recent technological advances in the assembly of functional hydrogel microstructures such as microfluidic, 3D bioprinting, and micromold-based 3D hydrogel fabrication methods have enabled the formation of 3D tissue constructs. However, they still lack flexibility and high efficiency, which restrict their application in 3D tissue constructs. Alternatively, we report a feasible method for the fabrication and reconstruction of customized 3D hydrogel blocks. Arbitrary hydrogel microstructures were fabricated in situ via flexible and rapid light-addressable electrodeposition. To demonstrate the versatility of this method, the higher-order assembly of 3D hydrogel blocks was investigated using a constant direct current (DC) voltage (6 V) applied between two electrodes for 20–120 s. In addition to the plane-based two-dimensional (2D) assembly, hierarchical structures—including multi-layer 3D hydrogel structures and vessel-shaped structures—could be assembled using the proposed method. Overall, we developed a platform that enables researchers to construct complex 3D hydrogel microstructures efficiently and simply, which has the potential to facilitate research on drug screening and 3D tissue constructs.

## 1. Introduction

The construction of a cell-friendly three-dimensional (3D) extracellular matrix (ECM) is a major challenge in the fields of biomedical and tissue engineering [1]. The 3D hydrogel-based tissue constructs, such as cellular microarrays and engineered tissue analogues, have been introduced as an alternative to animal experiments for advanced biomedical studies in vitro, pharmacological assays, observation of dynamic cellular process, and tissue morphogenesis using smaller sample volumes [2,3,4]. In order to generate geometry-controllable 3D hydrogel constructs, microfluidic technologies and 3D printing tools are widely applied. Currently, a variety of hydrogel shapes of particles, fibers, and sheets are used to reconstruct complex 3D tissue scaffolds in a bottom-up approach [5,6,7,8,9,10,11].

Artificial reconstruction is a very important strategy to achieve a higher-order assembly of several functional 3D tissue constructs. To implement this strategy, a number of emerging methods related to 3D hydrogel construction are available [12,13,14]. The micromold-based 3D hydrogel fabrication has high reproducibility due to the simple fabrication process. A number of 3D hydrogel constructs are easily formed in a 3D mold for use in high-throughput drug screening. Nevertheless, one substantial drawback of this method is a lack of flexibility of mold structure [15]. The microfluidic and 3D bioprinting technologies are efficient ways to fabricate 3D hydrogel constructs without the use of a mold. By using a combination of microfluidic and 3D bioprinting technologies, hydrogel blocks, capsules, and microfibers can be fabricated rapidly [5,6,7,8,9,11,16]. However, after the basic hydrogel blocks are constructed, it may be more difficult to reconstruct them into complex 3D tissues or multi-tissue architectures (e.g., blood vessels-shaped).

Recently, a shape control technique of 3D hydrogel construction based on electrodeposition was reported [17,18,19]. Because of its good biocompatibility and in situ cross-link with the calcium ions (Ca^2+^), Ca-alginate hydrogel has been widely applied to entrap and immobilize cells for the construction of 3D cellular tissue [3,20,21,22]. By means of electrodeposition, the 3D hydrogel patterns are generated on a 2D microelectrode surface, based on which in-situ 3D gel structures are fabricated with controllable size and shape. It is promising to fabricate the 3D gel structures rapidly independent of the 3D mold. Nevertheless, the electrode patterns still require pre-fabrication via mask microfabrication methods, such as photolithography and wet etching techniques [23]. Once a given geometry is fabricated, the resulting pattern is fixed as well. Hence, a more flexible and simple 3D gel fabrication strategy is essential for efficient 3D tissue reconstruction.

In this paper, we report an easy-to-use and universal approach for the flexible and rapid fabrication of 3D hydrogel blocks of Ca-alginate and readily assemble them into multi-layer or planar welding tissue constructs. In contrast to our previous efforts [12,13,14,15], a mold-free light-addressable electrodeposition method was adopted for this work. The method enables the formation of complex 3D hydrogel constructs via organic photoconduction-based controllable 3D hydrogel patterning. Herein, a photoconductive chip was developed based on titanium oxide phthalocyanine (TiOPc) due to its broad absorption from visible to infrared regions [24]. The TiOPc layer allows the impedance to be tuned by illumination. Therefore, coating TiOPc on indium tin oxide (ITO) glass yields a virtual electrode. Consequently, the electrode pattern is controlled via a visible-light projection onto the TiOPc chip. In this work, a Ca-alginate solution—a biocompatible hydrogel—was used to format the 3D tissue constructs. A programmable 3D hydrogel micropattern was fabricated using custom-designed optical patterns and a direct current (DC) electric field, which enabled Ca^2+^ cross-linking with alginate to form a 3D hydrogel microstructure in situ. The method provides a more effective fabrication platform for the 3D tissue constructs’ assembly into multi-layer or planar welding 3D bioconstructs, which has the potential to promote research into cell interaction mechanisms, single cell culture, and tissue reconstruction.

## 2. Materials and Methods

### 2.1. Chip Design and Fabrication

**Figure 1:**
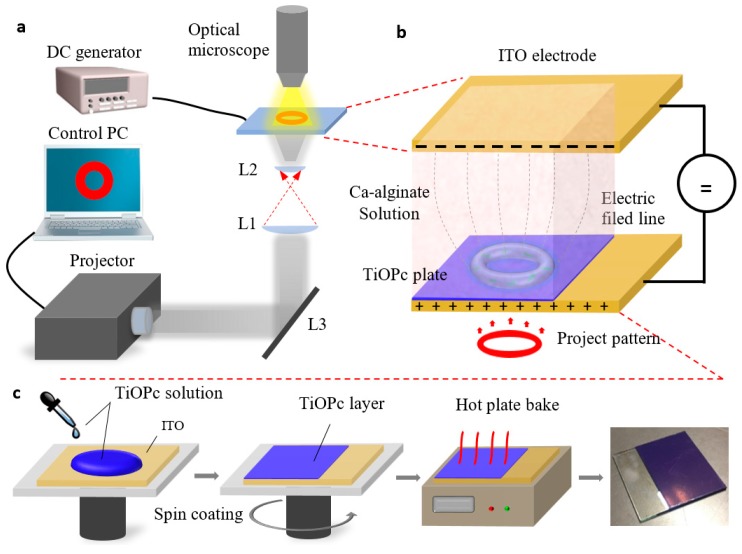
Schematic of the rapid light-addressable system and preparation process of the titanium oxide phthalocyanine (TiOPc) plate. (**a**) The programmable light patterns are projected onto the photoconductive chip through contraction lenses L1 and L2. The 3D hydrogel fabrication processes was observed under an optical microscope with a 10× objective lens and a charge-coupled device (CCD) camera. (**b**) The top indium tin oxide (ITO) glass and bottom TiOPc plate represent the two electrodes of the photoconductive chip. During the electrodeposition process, a Ca-alginate solution was introduced between the two electrodes. A DC electric field and an optical pattern triggered Ca-alginate solution cross-linking to generate 3D hydrogel constructs based on the virtual electrode pattern. (**c**) A TiOPc layer was generated by spin-coating at a certain spinning speed. Its thickness can be modified by adjusting the spinning speed.

First, 500 μL TiOPc solution was dropped onto the top surface of a 30 mm × 30 mm indium tin oxide (ITO) glass, which was spun at a speed 500 rpm for 15 s and accelerated at a speed 1200 rpm for 60 s, resulting in a layer of approximately 10 μm. Next, the coated plate was baked at 120 °C for 30 min to harden the TiOPc layer. The fabrication process is illustrated in Figure 1c.

The photoconductive chip was composed of three parts: a top ITO glass served as one of the electrodes, a bottom ITO glass surface coated with a thin layer of TiOPc as a light-addressable electrode, and a Ca-alginate hydrogel as an electrically-induced deposition solution. It represented a sandwich structure, as shown in Figure 1b. The distance between the two electrodes was adjusted according to the height of the fabricated 3D hydrogel structures from 40 μm to 1500 μm.

### 2.2. Preparation of Deposition Solution

Sodium alginate powder (Sigma Inc., Marlborough, MA, USA) was dissolved in distilled water at 1% (*w*/*v*). Then, insoluble CaCO_3_ powder (diameter 30–50 nm; Haofu Chemistry Limited Company, Shanghai, China) was dispersed into the solution at 0.5% (*w*/*v*) followed by magnetic stirring at 1200 rpm for 12 h.

### 2.3. Fluorescent Imaging

To obtain the fluorescent images of 3D hydrogel structures, 1% (*w*/*v*) suspension of the fluorescent microspheres (10 μm in diameter, Aladdin Inc., Shanghai, China) was mixed with the deposition solution and sonicated for 10 min. A fluorescence microscope (Eclipse Ni, Nikon, Tokyo, Japan) was used to observe the stained hydrogel at 4× magnification.

### 2.4. Formation of the 3D Hydrogel Microcapsule

To prevent the breaking of the hydrogel during reconstruction, the alginate-PLL (Poly-l-Lysine)microcapsule was fabricated as a shell. After the fabrication of the 3D hydrogel structures, the TiOPc plate was immersed into a 10 cm petri dish to flush away extra deposition solution. Then, the 3D hydrogel structures were detached from the TiOPc plate surface and transferred into another petri dish filled with 0.05% (*w*/*v*) PLL solution by gentle pipetting. The Ca-alginate hydrogel structures reacted with the PLL solution for 5 min, and the alginate–PLL membrane was formed. Next, 1.1% (*w*/*v*) CaCl_2_ solution was used to harden the alginate–PLL gel structures. The hardened 3D hydrogel structures were then treated with 0.03% (*w*/*v*) sodium alginate solution for 4 min to obtain the final microcapsule shell.

## 3. Results

For electrodeposition of Ca-alginate hydrogel, a rapid light-addressable system was used as shown in Figure 1a. The illumination source was provided by a projector. Then, the optical patterns programmed via a commercial computer were projected onto the TiOPc light-addressable electrode. The optical image contraction system consisted of two lenses of focal lengths (L1 and L2) 200 mm and 20 mm, respectively, and a dichroic mirror (L3), which served to minimize the optical pattern 1/10×. A DC potential applied between two electrodes was supplied by a signal generator (2601B, Keithley, Cleveland, OH, USA). The photoconductive chip was placed on a 3D translation platform, and the 3D hydrogel microstructures fabrication processes were monitored under an optical microscope with 10× objective lens and a charge-coupled device (CCD) camera.

**Figure 2:**
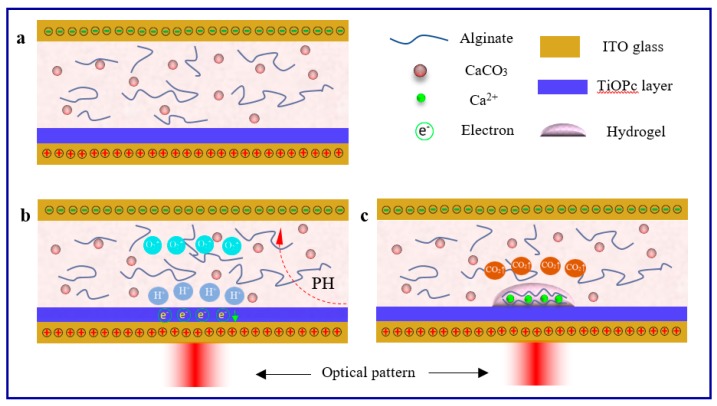
Schematic of light-induced electrodeposition. (**a**) The Ca-alginate solution was introduced between the cathodic ITO glass and the anodic TiOPc plate. (**b**) Light and DC voltage triggered the production of H^+^ nearby the anode, leading to a decreased pH gradient. (**c**) H^+^ reacted with CaCO_3_ nanoparticles in the solution to release Ca^2+^. After that, alginate cross-linking with the release of Ca^2+^ formed the 3D hydrogel structures based on the virtual electrode patterns.

The procedure of optically-induced 3D Ca-alginate hydrogel electrodeposition is illustrated in Figure 2. As mentioned earlier, the impedance of TiOPc could be tuned by illumination. Hence, the TiOPc layer was insulated when no light was projected on it, even when the DC source was applied between the two electrodes. As a consequence, electrically-induced deposition could not be triggered, as shown in Figure 2a. Whereas, after the optical pattern was projected on the TiOPc electrode, the impedance was adjusted by the projected area and the TiOPc electrode was conductive immediately. The photoconductive chip formed into a circuit, and the Ca-alginate hydrogel electrodeposition triggered on the positive electrode. Herein, a DC voltage of constant potential (6 V) was applied between the two electrodes for 20–120 s. The electric field across the solution generated H^+^ by electrolysis of water and formed a pH gradient around the anode surface (TiOPc layer), as shown in Figure 2b.

(1) $$2H_{2}O\to O_{2}+4H^{+}+4e^{-}$$

After that, the nano CaCO_3_ particles in the solution reacted with H^+^ to release calcium ions (Ca^2+^). 

(2) $${2H}^{+}{+\mathrm{CaCO}}_{3}\to H_{2}{O+\mathrm{Ca}}^{2+}{+\mathrm{CO}}_{2}$$

Finally, alginate in the solution cross-linked with the released Ca^2+^ to form the 3D Ca-alginate hydrogel structure onto the TiOPc plate corresponding to the virtual electrode pattern (Figure 2c).

(3) $${\mathrm{Ca}}^{2+}{+2\mathrm{Alg-COO}}^{-}\to \mathrm{Alg-COO-Ca-OOC-Alg}$$

**Figure 3:**
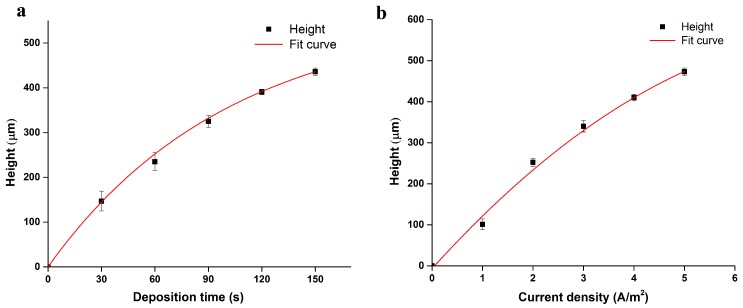
(**a**) The dependence of the deposition time on the height of the gel growth at constant current density 3 Am^−2^. The root mean squared error (RMSE) between experiment and model is 5.9 μm. (**b**) The effect of the current density on the hydrogel growth. The RMSE between experiment and model is 0.1 μm.

During the growth of the Ca-alginate hydrogel by electrodeposition, the density of Ca^2+^ was increased near the anode surface during the electrodeposition process as the time increased. Likewise, the gel’s height and hardness increased with time, and eventually reached a steady state. Thus, the formation of the Ca-alginate hydrogel is related to current density and period of illumination. The relationship between them has been exhibited in Figure 3. The dependence of deposition time on the height of gel growth is illustrated in Figure 3a. The trend of the gel growth increased linearly in time and finally reached approximately 400 µm at the constant current density of 3 Am^−2^. The effect of current density on hydrogel growth at the constant deposition time of 90 s was measured, which is shown in Figure 3b. The fit curve clearly confirms the relationship between the current density and the height of gel growth; i.e., the hydrogel grew faster under a higher current density.

**Figure 4:**
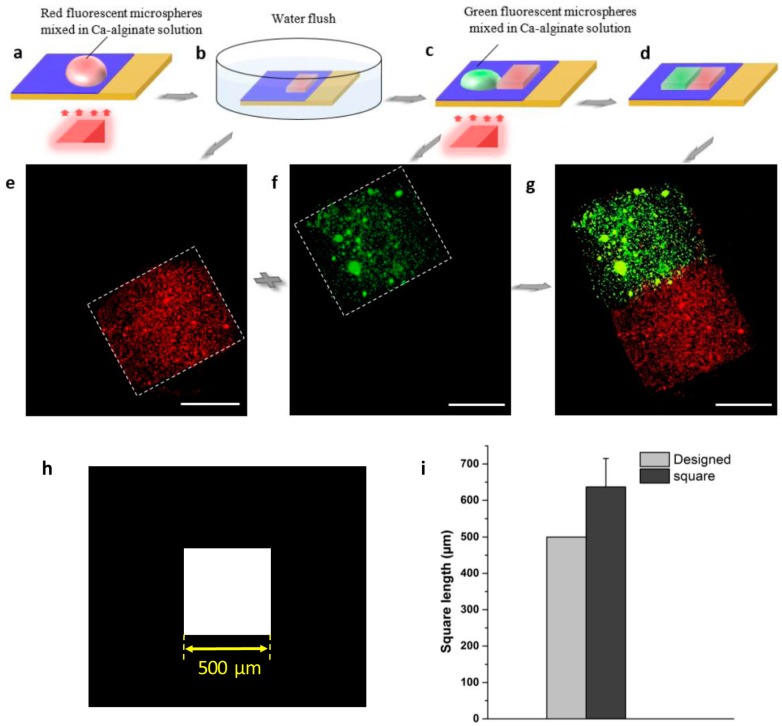
Reconstruction of 3D hydrogel blocks via planar welding. (**a**) The 3D hydrogel microstructure (red-square) was fabricated via a light-addressable electrodeposition system. (**b**) The 3D hydrogel structure came out after the deionized water gently flushed away the extra solution. (**c**) The Ca-alginate solution mixed with green fluorescent microspheres was dropped on the TiOPc layer adjacent next to the red-square 3D hydrogel structure, and the same hydrogel fabrication procedure was applied on it again. (**d**) After 50 s, the full-rectangle 3D hydrogel structure was achieved via planar welding of the green-square 3D hydrogel block with the red one. (**e**–**g**) The fluorescence images of the planar-weld 3D hydrogel structure. All scale bars are 250 µm. (**h**) The designed patterns of the virtual electrode. (**i**) Size distribution of designed pattern and 3D hydrogel microstructure.

To demonstrate the capability of the 3D hydrogel microstructures assembly achieved by this method, the three main types of 3D hydrogel construction assembly strategies were investigated systematically. The plane-based two-dimensional (2D) assembly is the common artificial construction strategy. The process of reconstructing two squared 3D hydrogel blocks into a full rectangular 3D hydrogel structure by planar welding is depicted in Figure 4. Red and green fluorescent microspheres were mixed into the Ca-alginate solution respectively for distinguishing the different 3D hydrogel blocks. A square optical pattern was projected on the TiOPc plate, and a red, squared 3D hydrogel block was fabricated via the light-addressable electrodeposition firstly, as shown in Figure 4a. After 50 s, the TiOPc plate was immersed into a petri dish (50 cm diameter) containing deionized water to gently flush away the extra alginate, and the boundary of the squared 3D hydrogel structure was defined clearly (Figure 4b). Again, a Ca-alginate solution mixed with green fluorescent microspheres was dropped on the TiOPc plate and joint with the red-square 3D hydrogel block. A squared optical pattern was projected on the TiOPc plate close to or slightly overlying the area of the red-squared 3D hydrogel block (Figure 4c). The same 3D hydrogel block fabrication process was carried out for the green-squared 3D hydrogel block, and a full rectangular 3D hydrogel structure was obtained via planar welding of two 3D squared hydrogel blocks (Figure 4d). Figure 4e–g show the assembly of the two 3D squared hydrogel blocks successfully. To avoid breaking the fragile hydrogels, an alginate–PLL membrane was formed as a shell to wrap the 3D hydrogel structure, sequentially forming a hydrogel microcapsule.

The photoconductive electrodes allowed the Ca-alginate hydrogel to form into a desired pattern at a specific address. To demonstrate the flexibility and reliability of this 3D gel microstructure fabrication strategy, various 3D hydrogel blocks were fabricated according to the virtual electrode patterns at will and were then assembled into the desired 3D tissue constructions in the condition of constant potential voltage 6 V and deposition time 20–120 s. The method of reconstructing 3D hydrogel blocks into new 3D hydrogel architecture was developed by the light-addressable electrodeposition. Consequently, it is suggested that the present method will allow researchers to fabricate more complex 3D tissues in vitro (e.g., blood vessels, muscle fibers, and neural pathways). It is noteworthy that they are the hierarchical structures of the human body. 

**Figure 5:**
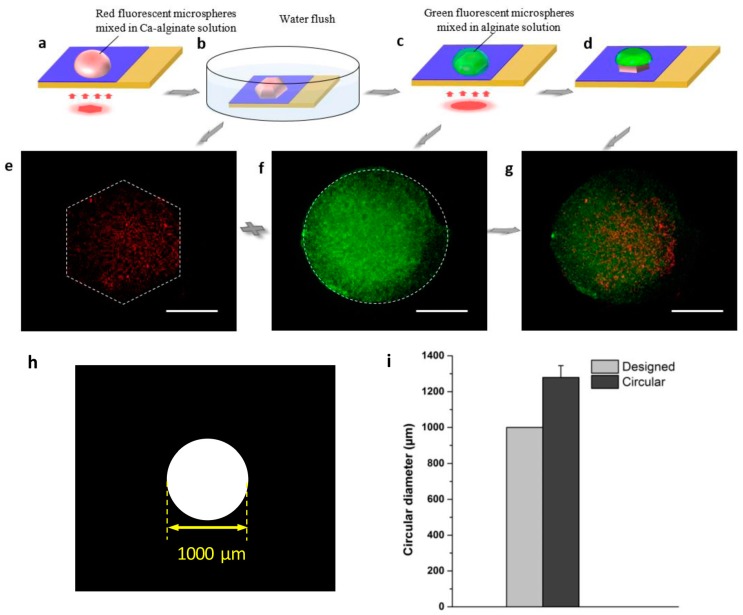
Formation of multi-layer 3D hydrogel microstructure. (**a**) A hexagonal optical pattern was projected on the TiOPc plate, and a red 3D hydrogel structure formed in the anode as the first layer. (**b**) After deposition, the deionized water was used to flush away the excess hydrogel solution. (**c**) Based on the first layer, the hydrogel solution without CaCO_3_ nanoparticles was dropped on it, and a circular optical pattern was projected on the overlying area to form a second layer. (**d**) A multi-layer 3D hydrogel microstructure assembled with the red hexagonal 3D hydrogel block and the green circular 3D hydrogel block. (**e**–**g**) The fluorescence images of the multi-layer 3D hydrogel structure. All scale bars are 250 µm. (**h**) The designed patterns of the virtual electrode. (**i**) Size distribution of designed pattern and 3D hydrogel microstructure.

Besides the planar welding, the hierarchical structures can be also assembled by the flexible and rapidly light-addressable electrodeposition method, which are useful for the creation of various complex functional objects, such as multi-layer 3D hydrogel structures and vessel-shaped structures. Figure 5 shows the building process of the multi-layer 3D hydrogel structures. A red-hexagonal 3D hydrogel structure was fabricated via light-addressable electrodeposition as the first layer, as shown in Figure 5a,b, and a 3D hydrogel structure based on a desired optical pattern was obtained, as shown in Figure 5e. For the second layer, green fluorescent microspheres were mixed with 1% (*w*/*v*) sodium alginate solution and dropped on the anode to overlay the area of the first layer, which serves as the feeder layer for the provision of free Ca^2+^. The first feeder layer contacts with the sodium alginate solution, then the anode current and optical pattern together trigger the Ca^2+^, which is released from the excess CaCO_3_ nanoparticles in the feeder layer to form the second layer of the Ca-alginate cross-linking hydrogel structures (Figure 5c,d). Herein, a circular optical pattern was projected on the overlying area as the second layer. After 60 s, a green-circular 3D hydrogel structure was generated on the first layer, and later the multi-layered structure was assembled successfully. The experimental results are shown in Figure 5f,g, respectively.

**Figure 6:**
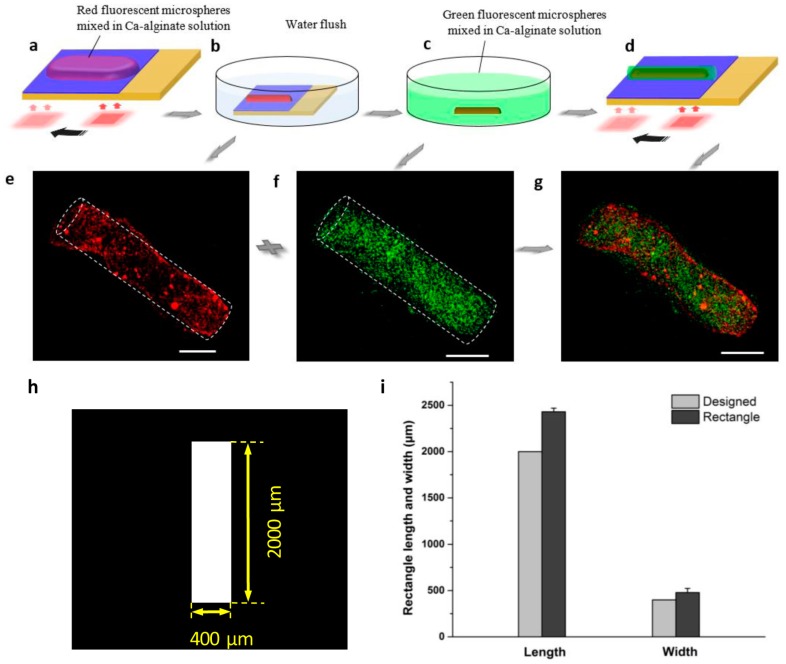
Assembly processes of the vessel-shaped structure. (**a**) The inner layer was fabricated via light-addressable electrodeposition step by step via optical pattern moved along the deposition solution cover area. (**b**) The TiOPc plate was immersed into the deionized water to flush away the extra deposition solution. (**c**) The formed inner layer was detached by pipetting gently and transferred into the sodium alginate solution mixed with green fluorescent microspheres. (**d**) The same fabrication steps were used to form the outer layer. (**e**–**g**) The fluorescence images of the vessel-shaped hydrogel structure. All scale bars are 400 μm. (**h**) The designed patterns of the virtual electrode. (**i**) Size distribution of designed pattern and 3D hydrogel microstructure.

The assembly process of the vessel-shaped structure is shown in Figure 6. To achieve a long and thin vessel-shaped 3D hydrogel structure, the optical pattern was projected on the TiOPc plate and moved along the Ca-alginate solution to cover the area step by step, with a deposition time of 50 s in each step. The consecutive electrodeposition was achieved as the designed length, the inner layer of the vessel-shaped structure was obtained as shown in Figure 6a. The fabricated inner layer can be clearly present via the deionized water flush away the extra Ca-alginate solution (Figure 6b). Herein, a squared optical pattern was used as the electrodeposition unit, and the length of the inner layer was approximately 2000 µm, while its width was approximately 400 µm. The fluorescence images of the vessel-shaped hydrogel structure are shown in Figure 6e. The fabrication process of the outer layer of the vessel-shaped structure is shown in Figure 6c,d. After flushing away the excess deposition solution, the inner layer 3D hydrogel structure was detached from the TiOPc plate by gentle pipetting and transferred into another 10 cm petri dish filled with the sodium alginate solution mixed green fluorescent microspheres. After treatment with the sodium alginate solution for 5 min, the fabricated inner layer was wrapped sufficiently. The wrapped inner layer was transferred to the TiOPc plate by pipetting gently. The same procedure as followed for the inner layer was used for the fabrication of the outer layer. After 50 s, an outer layer was fabricated with a length of approximately 2000 μm and width of approximately 450 μm. The results of the experiment are shown in Figure 6f. Figure 6g shows the successful assembly of the 3D vessel-shaped hydrogel microstructure. 

For this study, we fabricated various 3D hydrogel microstructures using this method. Figure 3 shows two square-shaped 3D hydrogel microstructures with length approximately 500 μm, while the length of the designed micro-pattern was 450 μm. The results indicate that the 3D hydrogel microstructures might be slightly larger than their corresponding designed micro-pattern because of the diffusion of calcium ions during the electrodeposition processes. We investigated the relationship between the designed optical pattern and the 3D hydrogel microstructures quantitatively. Three different micro-patterns were designed—squared with length 500 μm (Figure 4h), circular with diameter 1000 μm (Figure 5h), and rectangular with length 2000 μm and width 400 μm (Figure 6h), respectively. After light-induced electrodeposition, the length of the 2D square hydrogel microstructure was approximately 637 μm ± 78 μm (Figure 4i). The diameter of the 2D circular hydrogel microstructure was approxim0ately 1279 μm ± 67 μm (Figure 5i). The length and width of the 2D rectangular hydrogel microstructure were approximately 2430 μm ± 39 μm and 479 μm ± 45 μm (Figure 6i), respectively.

Consequently, the proposed method realized various 3D hydrogel blocks and the highly-efficient reconstruction of complex 3D microstructures. The advantage of this method is the capability to fabricate both plane-based 2D assembly and spatial assembly. Moreover, the structure and the shape of the 3D hydrogel constructs can be fabricated controllably (i.e., the length, width, thickness, and the layer numbers of hierarchical structures). The high flexibility and controllability of this method could have the potential to improve fabrication efficiency and the application of 3D hydrogel architectures.

## 4. Conclusions

In summary, we described a system for the study of the highly-efficient fabrication and flexible reconstruction of 3D hydrogel microstructures. The 3D hydrogel blocks can be fabricated readily and rapidly via mold-free light-addressable electrodeposition. The simple fabrication method presented herein allows the generation of desired hydrogel structures at a specific address. Furthermore, the method requiring only general electrodeposition and hydrogel fabrication processes can produce the construction of complex 3D hydrogel architectures without sophisticated techniques. Conclusively, the versa tile method holds metapromise as a universal platform for artificial and functional architectures applicable to drug screening and tissue transplantation.

## Supplementary Materials

The following are available online at www.mdpi.com/2072-666X/8/6/192/s1, Figure S1: The confocal fluorescence image of the 3D hydrogel microstructure. The scale bar is 200 μm.
