# Supplementary material for: Three-Dimensional Calcium Alginate Hydrogel Assembly via TiOPc-Based Light-Induced Controllable Electrodeposition
Source: Micromachines (Basel). 2017 Jun 19;8(6):192. doi: 10.3390/mi8060192 (PMC6189948; doi:10.3390/mi8060192)
Supplement: Supplementary file 1 [file micromachines-08-00192-s001.pdf]

Supplementary Materials: Three-Dimensional Calcium Alginate Hydrogel Assembly via TiOPc-Based Light-Induced Controllable Electrodeposition

Yang Liu, Cong Wu, Hok Sum Sam Lai, Yan Ting Liu, Wen Jung Li, Ya Jing Shen

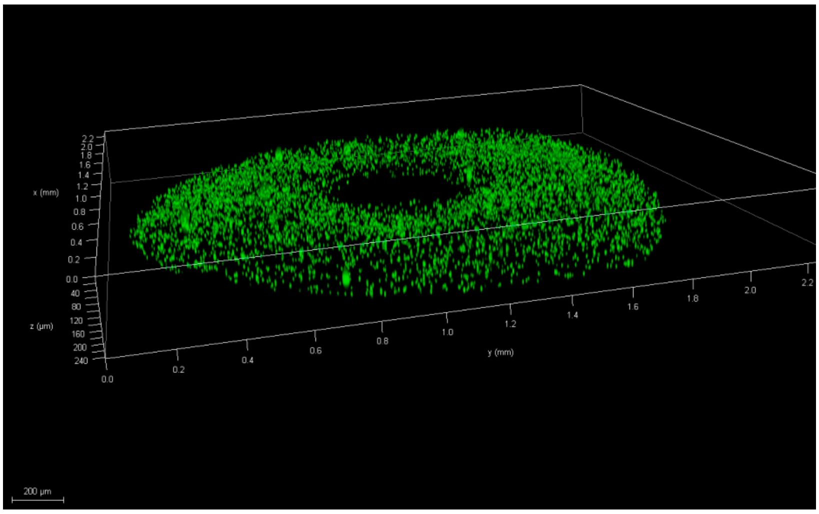

Figure S1. The confocal fluorescence image of the 3D hydrogel microstructure. The scale bar is 200 µm.
